# Breast Lymphoma: Teaching point: Primary breast lymphoma is a rare disease, especially in males, but should be considered in the differential diagnosis of a breast mass because of the different treatment and prognosis.

**DOI:** 10.5334/jbsr.1769

**Published:** 2019-04-09

**Authors:** Senne Jonckheere, Herman Depypere, Chloë Standaert

**Affiliations:** 1 UZ Gent, BE

**Keywords:** breast lymphoma, male, mammography, ultrasound, PET-CT

## Case

**Figure 1:**
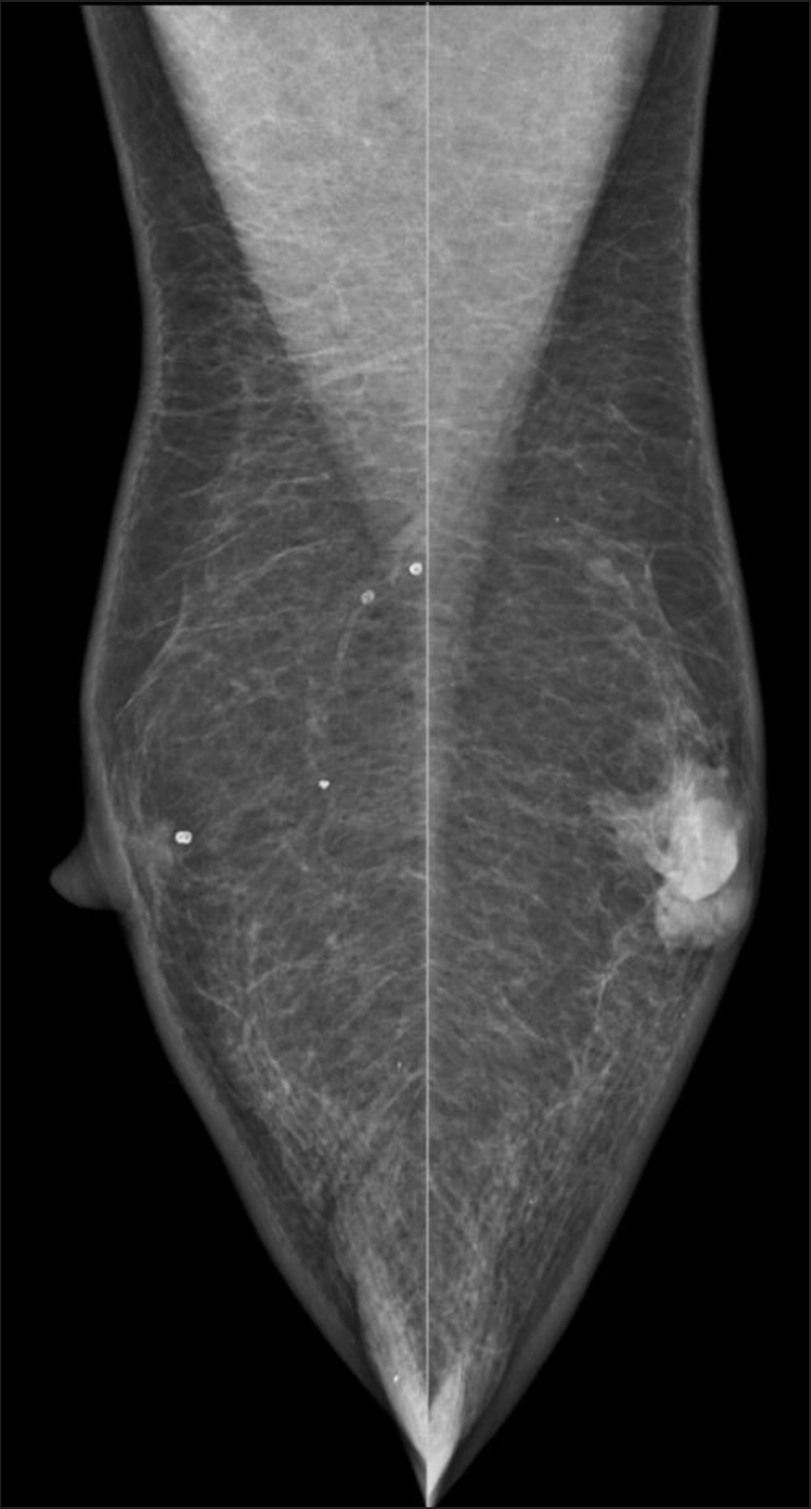
Retro-areolar mass in the left breast on mammography (oblique view).

**Figure 2:**
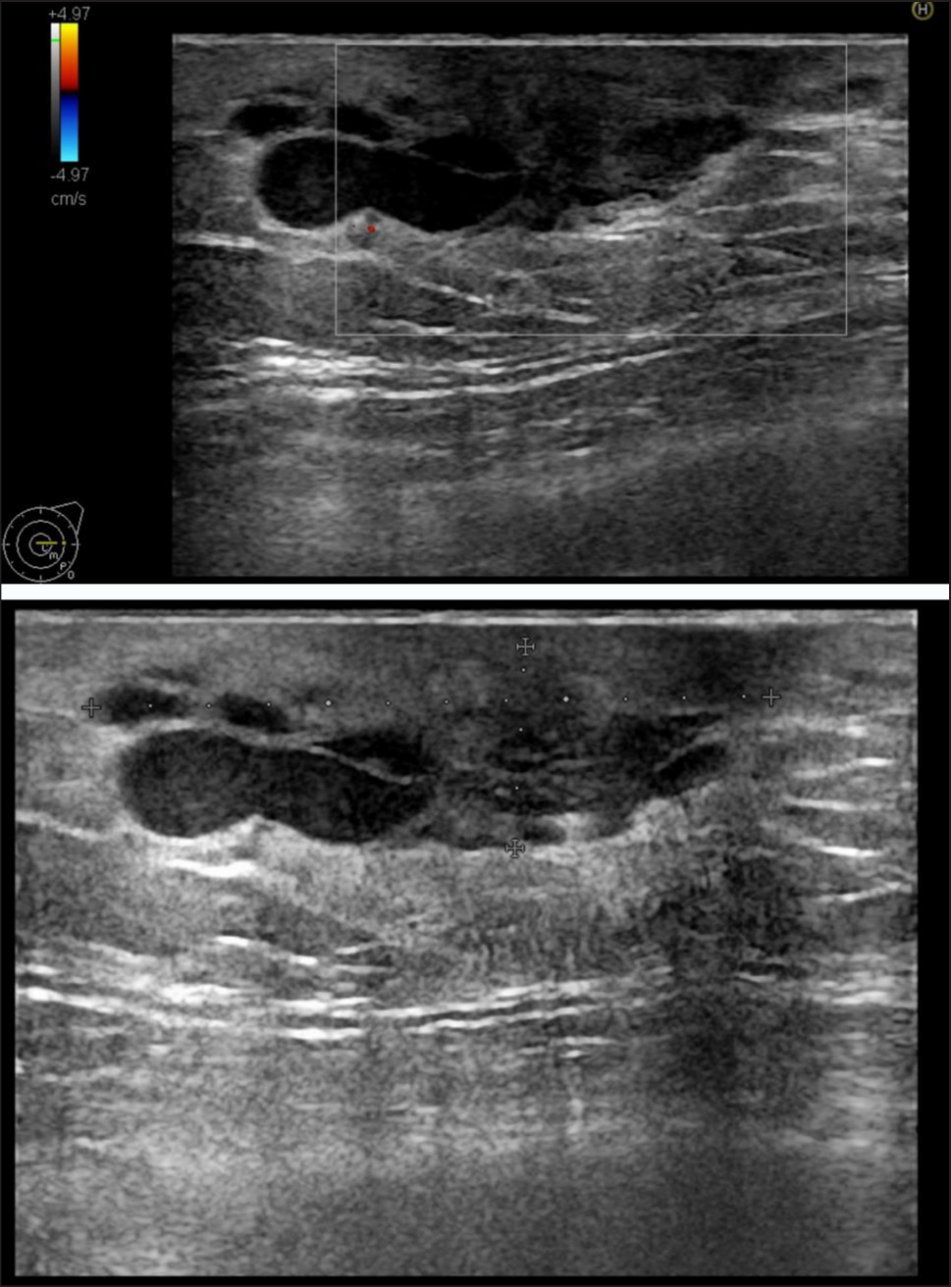
Retro-areolar hypo-echogenic mass with secondary mammary duct ectasia on ultrasound.

**Figure 3:**
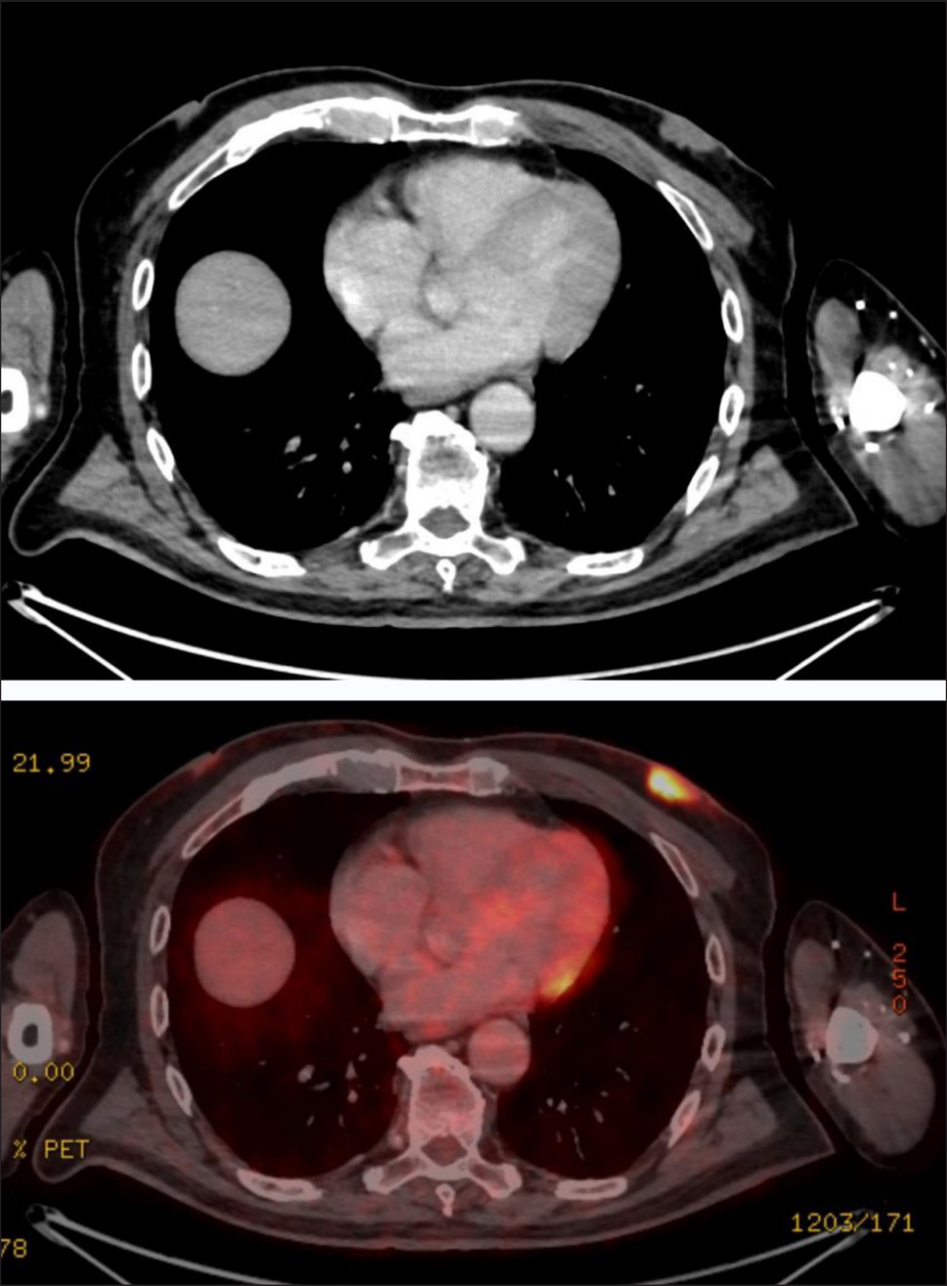
Metabolic active mass in the left breast on PET-CT.

An 80-year-old man was referred to the radiology department because of a persisting palpable mass in the left breast. Family history of breast cancer was negative. The mammography revealed a retro-areolar mass in the left breast (Figure 1). There were no microcalcifications. Ultrasound of the left breast showed predominantly enlarged mammary ducts, apparently caused by an ill-defined hypo-echogenic retro-areolar mass of 2.8 cm in diameter (Figure 2). An ultrasound-guided needle core biopsy of the retro-areolar mass was performed. Histology revealed a primary diffuse large B-cell type lymphoma of the breast. The patient had no prior history of lymphoma. Additional PET–CT showed a metabolic active lesion in the left breast (Figure 3). Widespread disease was not present.

## Comment

Primary breast lymphoma is a rare neoplasm of the breast with an incidence of less than 0.6% of primary breast malignancies. It is most frequently seen in the sixth decade. Due to the relatively small amount of lymphoid tissue in the breast, it has a low incidence and is even rarer in the male breast.

The clinical and histologic criteria for diagnosis of a primary breast lymphoma are the following: 1) a close association between breast tissue and infiltrating lymphoma; 2) no evidence of widespread lymphoma and no history of previous extramammary lymphoma; and 3) documentation of the breast as the principal organ involved and the primary site.

The differential diagnosis consists of invasive ductal carcinoma and metastatic disease to the breast. Invasive ductal carcinoma usually presents as a mass on imaging with the following features: microcalcifications, architectural distortion and spiculated margin. These features are rare in breast lymphoma. Metastatic disease of the breast was in our case less likely because there was no prior history of malignancy and the mass was single and unilateral. It is important to distinguish a breast lymphoma from breast cancer because of the different therapy (radiochemotherapy instead of surgery). Patients with breast lymphoma generally have a better prognosis than patients with breast cancer or extranodal lymphoma [1].

Breast lymphoma is a rare disease, especially in males, but it is important to differentiate it from breast cancer because of the different treatment and prognosis.

## References

[1] Rathod, J, Taori, K, Disawal, A, et al. A rare case of male breast lymphoma. J Breast Cancer. 2011; 14(4): 333–336. DOI: 10.4048/jbc.2011.14.4.333 22323922 PMC3268932

